# Identification of neurohypophysial hormones and the role of VT in the parturition of pregnant seahorses (*Hippocampus erectus*)

**DOI:** 10.3389/fendo.2022.923234

**Published:** 2022-07-29

**Authors:** Huixian Zhang, Yali Liu, Geng Qin, Qiang Lin

**Affiliations:** ^1^ Chinese Academy of Science (CAS) Key Laboratory of Tropical Marine Bio-resources and Ecology (LMB), Guangdong Provincial Key Laboratory of Applied Marine Biology, South China Sea Institute of Oceanology, Chinese Academy of Sciences, Guangzhou, China; ^2^ Southern Marine Science and Engineering Guangdong Laboratory (Guangzhou), Guangzhou, China; ^3^ University of Chinese Academy of Sciences, Beijing, China

**Keywords:** seahorse, vasotocin, male pregnancy, parturition, reproductive behavior

## Abstract

Neurohypophysial hormones regulate the reproductive behavior of teleosts; however, their role in the gestation and parturition of ovoviviparous fishes with male pregnancy (syngnathids) remains to be demonstrated. In the present study, the complementary DNA (cDNA) sequences of arginine vasotocin (VT) and isotocin (IT) from the lined seahorse (*Hippocampus erectus*) were cloned and identified. We observed that the mature core peptides of seahorse VT and IT were conserved among teleosts. In the phylogenic tree, seahorse VT and IT were clustered independently with teleost VT and IT. The tissue distribution patterns of *VT* and *IT* were similar, and both were highly expressed in the brain, gills, and gonads. Interestingly, they were also expressed to some extent in the brood pouch. *In situ* hybridization revealed that *VT* and *IT* messenger RNA (mRNA) signals in the brain were mainly located in the preoptic area region of the hypothalamus. Intraperitoneal administration of the VT core peptide to pregnant seahorses induced premature parturition, stimulated gonadotropin release, increased serum estrogen levels, and decreased prolactin secretion. Moreover, VT injection upregulated the mRNA expression of the membrane estrogen receptor in the brood pouch. In summary, neurohypophysial hormones promote premature parturition by regulating estrogen synthesis through the hypothalamus–pituitary–gonad axis.

## Introduction

Neurohypophysial hormones are hypothalamic neuropeptides transported to the posterior pituitary and function as the regulators of vertebrate reproduction. In mammals, neurohypophysial hormones, including oxytocin (OT) and arginine vasopressin (AVP), are primarily involved in uterine contraction, parturition, and lactation (1). OT and AVP only differ in two amino acids, which arose from a gene duplication event during evolution (2). In teleosts, isotocin (IT) is homologous to the mammalian OT whereas arginine vasotocin (VT) is homologous to AVP (3, 4). VT and IT are nonapeptide neurohormones that are primarily synthesized in the neurons of the hypothalamus. They play central roles in fish physiology and behavior, including reproduction, metabolism, osmoregulation, and social behavior (5, 6).

The administration of nonapeptide hormones affects the sexual behaviors and parturition of teleosts (7, 8). IT affects the ovulation of oviparous fishes, such as killifish (*Fundulus heteroclitus*), seurukan fish (*Osteochilus vittatus*), and Hoven’s carp (*Leptobarbus hoevenii*) (9–11). Similarly, VT injections stimulate the courtship behavior of the male white perch (*Morone americana*) (12) and influence the aggressive behavior of the rainbow trout (13). Moreover, VT can modulate steroidogenic shift and control serum E_2_ levels to stimulate oocyte maturation in female catfish (*Heteropneustes fossilis*) (14). In ovoviviparous fishes, the administration of IT and VT induces premature parturition in the guppy (*Poecilia reticulata*) and topminnow (*Gambusia affinis*) (15).

Syngnathids (pipefish and seahorse) are ovoviviparous fishes with male pregnancy and complex reproductive behavior (16). Syngnathid females transfer eggs into the brood pouch, which is a special structure on the ventral surface of males. Then, the eggs are fertilized and developed in the brood pouch until hatching. The brood pouch is similar to the mammalian placenta, and it can provide nutrition and oxygen to the embryos (17, 18). However, the endocrinological mechanism underlying the seahorse reproductive behavior, including male pregnancy and parturition, is still unknown.

In this study, we characterized the neurohypophysial hormones (VT and IT) of the lined seahorse (*Hippocampus erectus*) and detected their expression patterns in adult tissues. We also analyzed the locations of *VT* and *IT* genes in the brain. Finally, VT was injected *in vivo* to evaluate its functional role in the parturition of pregnant seahorses, its effects on serum sex steroid hormones, and the expression profiles of regulatory genes in the hypothalamus–pituitary–gonad (HPG) axis.

## Materials and methods

### Experimental fish and tissue sampling

Cultured adult lined seahorses (*H. erectus*) (6 months old) were collected from the Zhangzhou Fisheries Center, Fujian Province, China. Seahorses were maintained in seawater tanks at 26 ± 0.5°C, 26 ± 2% salinity, and a 12:12-h day-light cycle. The seahorses were fed twice daily with frozen Mysis shrimp and reared for 2 weeks before the experiments. The lined seahorses were anesthetized with 0.05% MS-222 (Sigma, St. Louis, MO, USA) and dissected. Tissues were collected, frozen in liquid nitrogen, and stored at an -80°C freezer until RNA extraction. All animal experiments were conducted in accordance with the guidelines and approval of the Animal Research and Ethics Committee of the Chinese Academy of Sciences.

### Cloning of the seahorse *VT* and *IT* genes

Total RNA was isolated from the brain using a TRIzol reagent (Invitrogen, Carlsbad, CA, USA), according to the manufacturer’s instructions. Total RNA (1 μg) was reverse-transcribed using the ReverTra Ace-α highly efficient reverse transcription kit (Toyobo, Osaka, Japan). Based on the brain transcriptome and genome data of the lined seahorse (19, 20), the full-length cDNA sequences of seahorse *VT* and *IT* were obtained using the rapid amplification of cDNA ends (RACE). The 5′-UTR and 3′-UTR ends were amplified using a SMARTer RACE 5′/3′ kit (Clontech, Palo Alto, CA, USA). The PCR primers used are listed in **Table 1**. Target fragments were subcloned into a PMD18-T vector (Takara, Dalian, China). The positive clones of target fragments were sequenced by Tsingke BioTech (Beijing, China).

**Table 1:** Information of primers used in this study.

Primer sequence			
Gene	Purpose	Primer	5’-3’ sequence
VT	Partial cDNA	VT_F1	GCTGATGCTCCTCCTGCTTGG
		VT_R1	AAGGCCGTTCTTCCGTAGTGC
	5’RACE	VT_R2 (first)	GTACGGGACGGTCACATAGCC
		VT_R3 (nest)	CTTCCACGAGGACCTCGTCTG
	3’RACE	VT_F2 (first)	GCTACTCGGCTCTGGGAAAGC
		VT_F3 (nest)	GTGACCGCCACGTCAGGATC
	Real-time PCR	VT_qF	CCTCCGCCTGCTACATCCAG
		VT_qR	CAGGGAGTCAGCAGGTAGTTC
IT	Partial cDNA	IT_F1	GCTGCAGCTCGGCACCTTCTGC
		IT_R1	GACGATTGATCCGAAGAACATC
	5’RACE	IT_R2 (first)	CACAGGCAGGACTGTGCGTTC
		IT_R3 (nest)	CATCTTCAGGATTGTCTCAGTG
	3’RACE	IT_F2 (first)	GGCTTCTACCACGAGCACAC
		IT_F3 (nest)	TCCACTGGGAGAACATTCAGG
	Real-time PCR	IT_qF	CCTTCCGCTTGCTGGTCT
		IT_qR	CAATGGGACAGTTGGAGATG
FSHβ	Real-time PCR	FSHβ_qF	GCAATGGGAACTGGACCTAC
		FSHβ_qR	TGATTGATACGAGCAGCACA
LHβ	Real-time PCR	LHβ_qF	CCAATAAGGTGCCAGGATGT
		LHβ_qR	ACCTGGAAGGCAGTTAGACA
PRL	Real-time PCR	PRL_qF	TGACGTCGGTCAGGACAAGC
		PRL_qR	CTTCAGCACAAGTGAGGTTGC
ERα	Real-time PCR	ERα_qF	TTACTCACCAGCATGGCTGAC
		ERα_qR	CCTCGGACTTGAGTCTGAGC
ERβ	Real-time PCR	ERβ_qF	GTCCTCACACAGCAAGACTC
		ERβ_qR	TCCAGAAGACTGAGCTCCAC
GPER	Real-time PCR	GPER_qF	CGTCTTCATCAGCATCCAGC
		GPER_qR	ACCGAAGGTCCCAAATGGAG
β-actin	Real-time PCR	β-actin_qF	TTCACCACCACAGCCGAGA
		β-actin_qR	TGGTCTCGTGGATTCCGCAG

### Phylogenetic and synteny analysis of seahorse vasotocin and isotocin

The cDNA sequences of seahorse *VT* and *IT* were translated into amino acid sequences by using Expasy (http://web.expasy.org/protparam). Multiple sequence alignments were performed using the DNAMAN software. The signal peptide was predicted using the SignalP 5.0 server (http://www.cbs.dtu.dk/services/SignalP/). A phylogenetic tree was constructed with Mega 6.0 software (21) using the neighbor-joining method (bootstrap = 1,000). A synteny analysis of *VT* and *IT* genes was performed by comparing the gene arrangements of the *VT* and *IT* gene loci in zebrafish, platyfish, tilapia, medaka, fugu, tiger-tailed seahorse, and lined seahorse. The gene loci of these teleosts were identified using the genome data from Ensembl (http://www.ensembl.org).

### Tissue distribution of seahorse vasotocin and isotocin

Total RNA was isolated from various tissues of male seahorses (n=3), including the brain regions (telencephalon, optic tectum, cerebellum, hypothalamus, and pituitary), liver, gill, intestine, kidney, muscle, heart, gonad, and brood pouch. Reverse transcription PCR was performed using 1 μg of RNA isolated from each tissue. PCR conditions were as follows: predenaturation at 94°C for 3 min; 30 cycles at 94°C for 15 s, 55°C for 15 s, 72°C for 30 s, and extension at 72°C for 10 min; and cooling to 4°C. The housekeeping gene (β-actin) was used as a positive control.

### RNA *in situ* hybridization

The fragments of *VT* and *IT* genes (528 and 589 bp, respectively) were subcloned into the pGEM-T easy vector. Plasmid DNA containing the *VT* and *IT* inserts was extracted using the E.Z.N.A Plasmid DNA Mini kit (Omega, Norcross, GA, USA). Sense and antisense riboprobes were synthesized from SalI- and NcoI-linearized pGEM-T easy plasmids using the DIG RNA Labeling kit (Roche, Mannheim, Germany). The brain samples of lined seahorse were fixed in 4% paraformaldehyde overnight at 4°C. The fixed brains were then dehydrated in 20% sucrose and embedded in an optimal cutting temperature (OCT) compound (Sakura, Tokyo, Japan). Cross-sections (10 μm), including those of the hypothalamus area, were collected on 3-aminopropylysilane-coated slides. Collected sections were digested with proteinase K (1 μg/ml) at 37°C for 20 min. The slices were prehybridized with hybridization buffer without probe at 58°C for 1 h and hybridized with DIG-labeled probes (0.5 μg/ml) in hybridization buffer at 58°C overnight in a sealed humidity chamber. After hybridization, slides were washed in 2× saline-sodium citrate (SSC) buffer and 0.1× SSC buffer for 30 min at 58°C. The slides were then blocked with 2% normal sheep serum and incubated with an alkaline phosphatase–conjugated anti-DIG antibody (Roche; 1:1,000 dilution). The color change reaction was conducted using an nitroblue tetrazolium (NBT)/5-bromo-4-chloro-3-indolye phosphate (BCIP) stock solution (Roche, Basel, Switzerland), and the reaction was stopped using tap water.

### *In vivo* effects of vasotocin on the parturition of pregnant seahorses

To evaluate the functional role of VT in parturition, pregnant seahorses were injected with [Arg^8^] vasotocin acetate salt (VT, V7009; Sigma). The pregnant seahorses (6 months old) were anesthetized and injected intraperitoneally with VT (high dose: 500 ng/g; low dose: 50 ng/g) and saline (n = 6) as the sham control. Seahorses from each group were sampled at 6-h postinjection. The pituitary and brood pouch were dissected and frozen in liquid nitrogen until RNA extraction. Blood samples were collected from the tail vessels at 6-h postinjection. Serum samples were separated by centrifugation at 3,000 × *g* for 30 min at 4°C and stored at -20°C. The serum levels of estrogen and testosterone were determined *via* an enzyme-linked immunosorbent assay (ELISA) kit (Cayman Chemical Company, Ann Arbor, MI, USA) (Estradiol ELISA kit no. 501890 and Testosterone ELISA kit no. 582701) according to the manufacturer’s protocol (22). Approximately 5 μl of serum sample was diluted in 45 μl of ELISA buffer to detect the concentration of estrogen and testosterone using the standard samples as a reference. The mRNA expression profiles of gonadotropins, follicle-stimulating hormone (FSHβ), luteinizing hormone (LHβ), and prolactin (PRL) in the pituitary were analyzed.

### Quantitative real-time PCR

The expression levels of all the tested genes were measured using the qRT-PCR methodology described previously (22). The primers used for the qRT-PCR assays are listed in **Table 1**. The template was amplified at 94°C for 1 min, followed by 40 cycles at 94°C for 15 s, 50−55°C for 15 s, and extension at 72°C for 20 s. The housekeeping gene β-actin was used as the reference gene. The relative abundance of each target gene was evaluated using the 2^−ΔΔCt^ method (23).

### Statistical analysis

Data were shown as the mean ± standard error of the mean (SEM). Significant differences were evaluated by one-way analysis of variance (ANOVA), followed by Duncan’s multiple range tests using Prism 6.0 (GraphPad Software, San Diego, CA, USA). Differences between groups with *p* < 0.05 were deemed significant.

## Results

### Characterization of seahorse *VT* and *IT* genes

**Figure 1:**
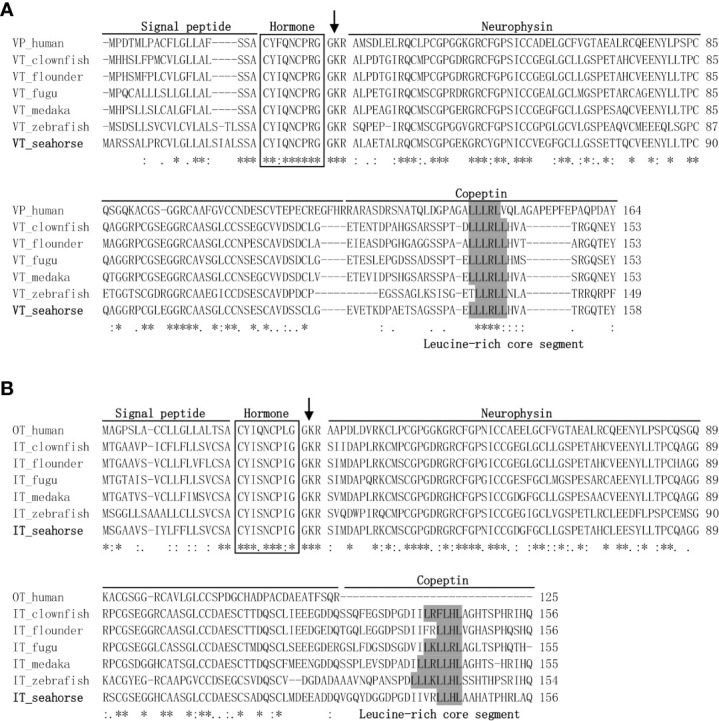
Genetic structure and multiple sequence alignments of vasotocin (VT) **(A)** and isotocin (IT) **(B)**. The mature hormone peptides are boxed, and the GKR cleavage site was marked with an arrow. Genetic structures, including a signal peptide, neurophysin, and copeptin parts, were lined out. GenBank accession numbers for teleost VT and IT proteins are listed in **Table 2**. Symbol asterisks means that the amino acid residues in that column are identical in the alignment.

The lined seahorse *VT* cDNA sequence (GenBank Accession No. MW490038) had an Open Reading Frame (ORF) of 477 bp, encoding for a protein of 158 amino acids (aa) (**Figure 1A**). The VT precursor had a signal peptide of 24 aa, a 9-aa hormone moiety (CYIQNCPRG), an enzymatic processing site [Gly-Lys-Arg (GKR)], and an 88-aa neurophysin. The *IT* precursor cDNA (GenBank Accession No. MW490039) had an ORF of 471 bp, encoding for a protein of 156 aa (**Figure 1B**). The IT precursor had a signal peptide of 19 aa, a hormone moiety of 9 aa (CYIQNCPLG), an enzymatic processing site, a 95-aa neurophysin, and a 30-aa copeptin region with a leucine-rich core segment. Homology analysis showed that seahorse VT had the highest sequence identity with the VT sequences of medaka (77.8%) and clownfish (77.8%) and that the IT sequence was highly similar to that of clownfish (80.1%) and flounder (78.8%) (**Table 2**).

**Table 2:** Similarities of amino acid sequences of the neurohypophysial hormone between the lined seahorse and other teleosts.

Species	Accession no.	Amino acid sequence identity (%)	
		VT	IT
VT_seahorse	MW490038	100.0	56.2
VT*_*clownfish	AEB00559.1	77.8	58.2
VT*_*flounder	BAA98140.1	75.8	58.2
VT_fugu	AAC60293.1	73.9	54.9
VT_medaka	BAM15897.1	77.8	56.2
VT_zebrafish	NP_840078.1	59.1	50.7
VP_human	NP_000481.2	52.0	45.2
IT_seahorse	MW490039	56.2	100.0
IT_clownfish	AEB00560.1	60.8	80.1
IT_flounder	BAA98141.1	58.2	78.8
IT_fugu	O42493.1	54.9	72.9
IT_medaka	BAM15898.1	55.9	75.5
IT_zebrafish	AAL50209.1	49.3	59.5
OT_human	NP_000906.1	54.4	50.4

### Phylogeny and synteny analyses of seahorse vasotocin and isotocin

**Figure 2:**
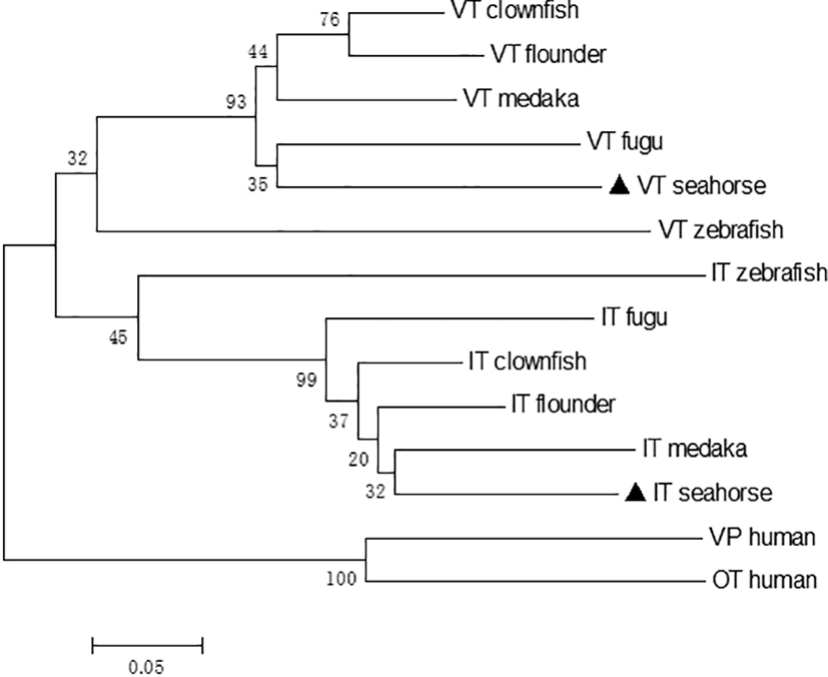
Neighbor-joining phylogenetic tree of VT and IT and their homologous sequences in vertebrates. The numbers indicate the bootstrap values of 1,000 replicates.

The phylogenetic tree revealed that the neurohypophysial hormones were clustered into two separate clades: VT and IT clades. Seahorse VT were clustered together with other teleost VT precursors, while seahorse IT were clustered with other teleost IT precursors (**Figure 2**). Human vasopressin (VP) and OT were used as outgroups. A synteny analysis of *VT* and *IT* showed that the organization and orientation of these genes in the loci were highly conserved among teleost species (**Figure S1**). The synteny of *VT* and *IT* in seahorse was the same as that in other teleosts, except for zebrafish, in which *VT* and *IT* were located on two separate chromosomes.

### Tissue distribution of seahorse vasotocin and isotocin

**Figure 3:**
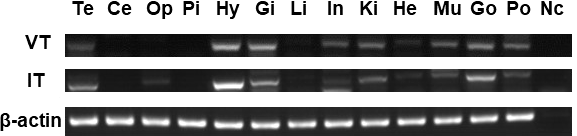
Analysis of *VT* and *IT* expression patterns in various tissues of male seahorses by reverse transcription PCR. The brain regions: Te, telencephalon; Ce, cerebellum; Op, optic tectum; Pi, pituitary; and Hy, hypothalamus. The peripheral tissues: Gi, gill; Li, liver; In, intestine; Ki, kidney; He, heart; Mu, muscle; Go, gonad; Po, brood pouch. Nc, negative control.

The tissue distribution of seahorse *VT* and *IT* was determined using semiquantitative reverse transcription PCR (**Figure 3**). Both *VT* and *IT* mRNAs were widely expressed in the central nervous system and peripheral tissues and were highly expressed in the hypothalamus, gills, and gonads. Interestingly, these genes were also expressed in the brood pouch to some extent.

### Localization of vasotocin and isotocin in the brain

**Figure 4:**
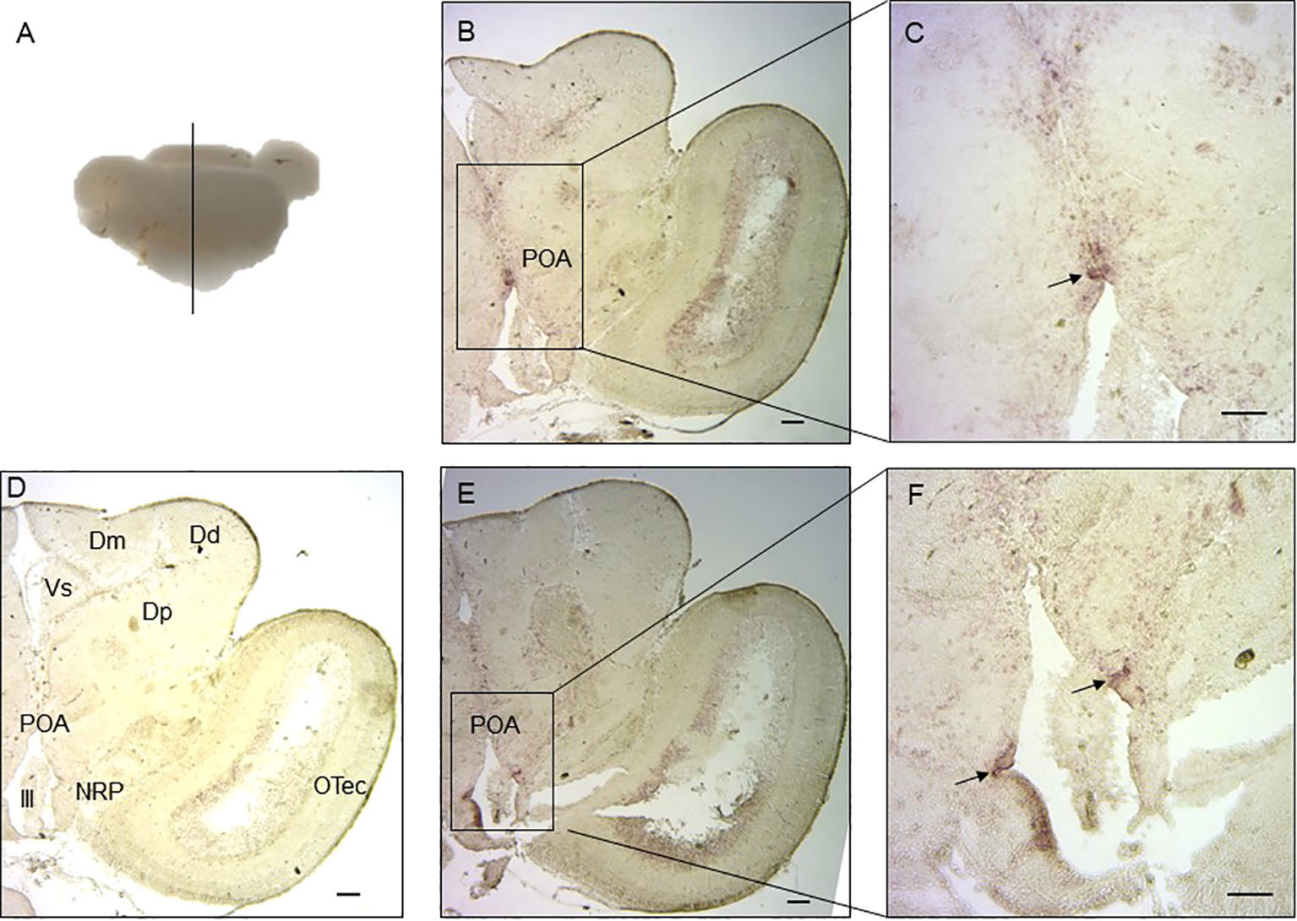
Localization of cells expressing *VT* and *IT* mRNAs in the lined seahorse brain. **(A)** Schematic diagram indicating the position of the coronal section and brain slides selected in the following. **(B)** *In situ* hybridization with an antisense *VT* mRNA probe showing the midbrain coronal section. **(C)** Enlarged part of the hypothalamus region using a *VT* antisense probe. **(D)** *In situ* hybridization with a sense *VT* probe within hypothalamus and midbrain regions of the lined seahorse. The brain regions are marked out in the micrograph. **(E)**. *In situ* hybridization with an antisense *IT* mRNA probe showing the midbrain coronal section. **(F)** Enlarged part of the hypothalamus region using and *IT* antisense probe. OTec, optic tectum; Dd, dorsal part of dorsal telencephalon; Dp, posterior part of dorsal telencephalon; Dm, medial part of dorsal telencephalon; Vs, supracommissural nucleus of ventral telencephalon; POA, preoptic area; NRP, the nucleus of posterior recess; III, the third ventricle. Scale bars = 200 μm.

The functions of lined seahorse VT and IT were evaluated based on their relative expression patterns. Therefore, the localization of the cells expressing *VT* and *IT* mRNAs was detected in the lined seahorse brain (hypothalamus) *via in situ* hybridization. Both *VT* and *IT* mRNAs were located in the preoptic area (POA) region of the hypothalamus (**Figure 4**). *VT* mRNA was mainly expressed in the central area of the POA region, whereas *IT* mRNA was expressed in the edge area of the POA region.

### Effects of vasotocin administration on the parturition of pregnant seahorses

**Figure 5:**
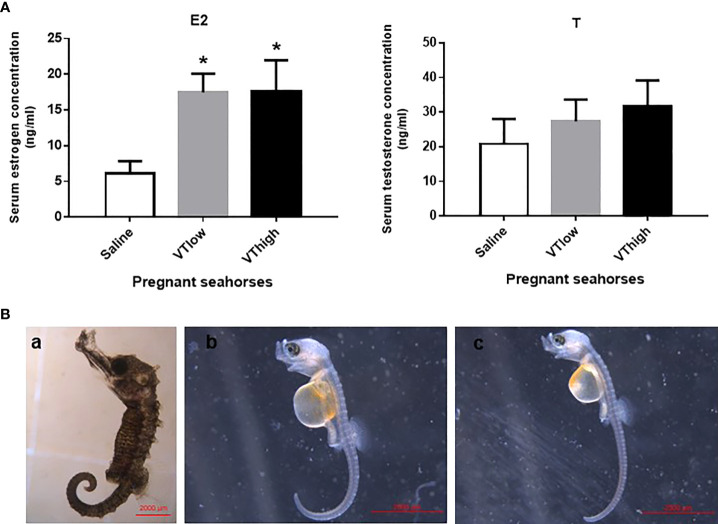
Serum estrogen and testosterone levels after VT injection **(A)**. Seahorse larvae injected with low **(B-b)** and high doses **(B-c)** of VT compared to normal hatched seahorse larvae **(B-a)**. Data are presented as mean ± SEM. Asterisks indicate significant differences between different treatments (*p* < 0.05).

**Figure 6:**
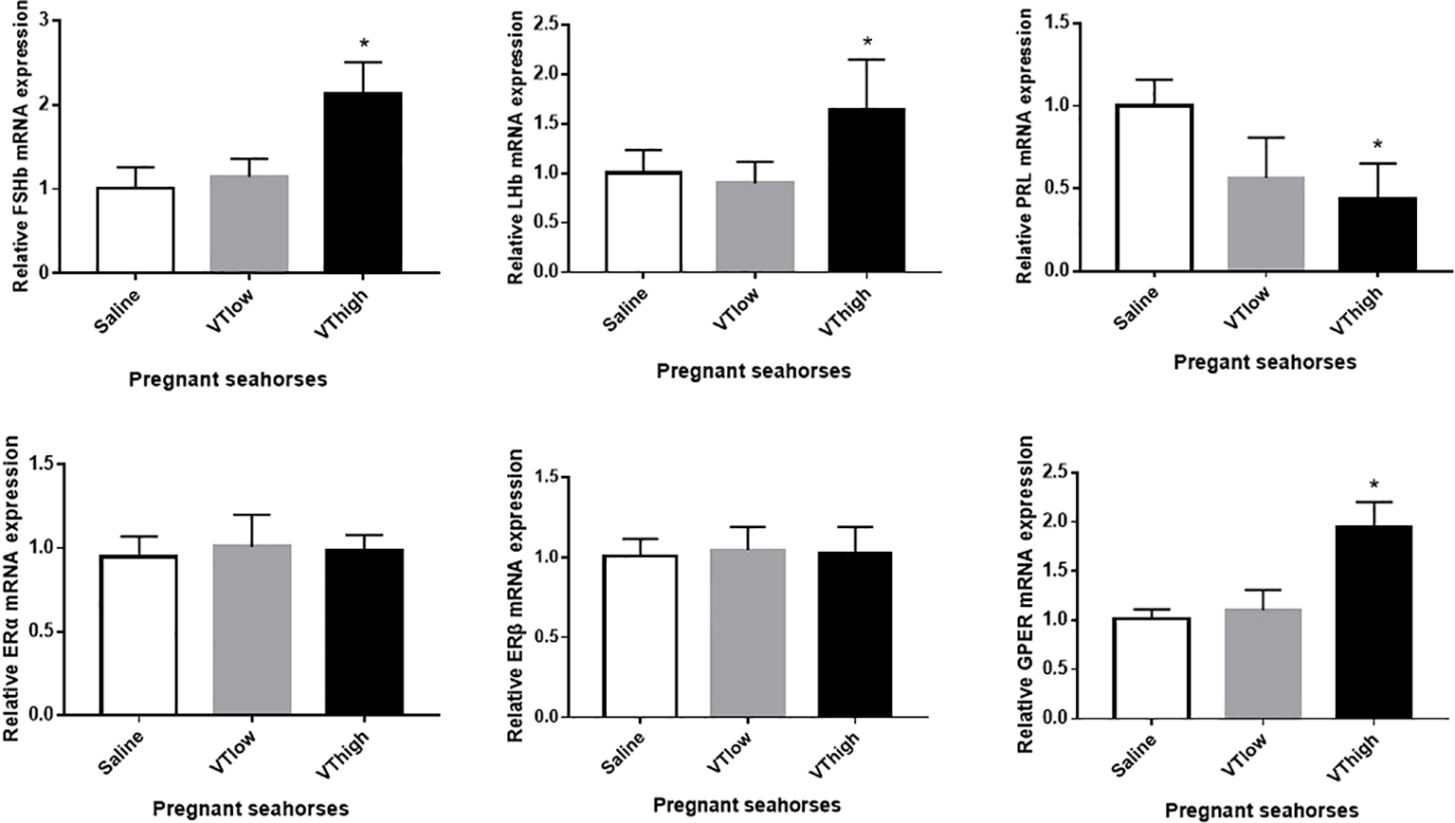
Effects of the VT core peptide injection on the expression of *FSHβ*, *LHβ*, and *PRL* genes in the pituitary and *ERα*, *ERβ*, and *GPER* genes in the brood pouch of pregnant lined seahorses. The mRNA expression levels were determined using qRT-PCR. Data are presented as mean ± SEM. Asterisks indicate significant differences between different treatments (*p* < 0.05).

After VT injection, serum estrogen levels increased remarkably, whereas serum testosterone levels did not change significantly (**Figure 5A**). The pregnant seahorses continued swinging their body to perform parturition behavior to let the seahorse larvae hatch out from the brood pouch. In addition, premature seahorse larvae (induced by high and low doses of VT) still had large yolk sacs and were shorter than the larvae that hatched normally (**Figure 5B**, **Table 3**). *FSHβ* and *LHβ* mRNA levels increased significantly, whereas *PRL* mRNA levels decreased significantly in the pituitary of pregnant seahorses. Moreover, the mRNA expression of the membrane estrogen receptor [G protein-coupled estrogen receptor (*GPER*)] in the brood pouch significantly increased after a high-dose VT injection, whereas *ERα* and *ERβ* mRNAs showed no significant changes (**Figure 6**).

**Table 3:** Effect of the AVT hormone on parturition in the lined seahorses.

Dose (ng/g body weight)	Total no. of seahorses	Standard length of larvae (mm)	Yolk sac diameter (mm)
0 (Saline)	6	7.82 ± 0.02	<0.05
50 (Vasotocin)	6	6.73 ± 0.05*	0.94 ± 0.03*
500 (Vasotocin)	6	6.24 ± 0.03*	0.85 ± 0.02*

Values shown are means ± standard errors. *Significantly (P < 0.05) different from control.

## Discussion

In the present study, the cDNAs of the neurohypophysial hormones *VT* and *IT* were cloned and characterized in the lined seahorse. Sequence analysis revealed that the mature peptides of VT and IT were highly conserved among the teleost species. In addition to the signal and mature peptides, the neurohypophysial nonapeptide precursor had three domains: GKR, neurophysin, and copeptin (2, 24). The GKR domain, which is involved in the cleavage and amidation of the hormone, was conserved. However, the N-terminus of neurophysin was divergent whereas the rest was conserved. The domains of neurohypophysial hormones in teleosts are identical (3). Phylogeny analysis revealed that seahorse VT was clustered with other teleost VTs, and seahorse IT was clustered with other teleost ITs independently. Synteny analysis showed that *VT* and *IT* gene loci were conserved among teleosts. These results showed that the cloned seahorse *VT* and *IT* genes are orthologous to those of other teleosts.

Reverse transcription PCR showed that both *VT* and *IT* mRNAs are highly expressed in the hypothalamus, gills, and gonads of the lined seahorse, similar to the findings in other teleosts (25, 26). Seahorse *VT* mRNA was expressed much more widely than *IT* mRNA, suggesting that VT plays more diverse roles in various tissues than IT. The high expression in sexual tissues suggests that VT and IT play important roles in regulating reproductive behavior. In addition, the expression in the gills suggests that VT and IT are involved in osmoregulation (5).

*In situ* hybridization showed that seahorse *VT* and *IT* were mainly expressed in the cells of the POA region in the hypothalamus. This result is consistent with the findings in the orange-spotted grouper (27), bluehead wrasse (7), and weakly electric fish (28). Most studies in teleosts have suggested that *VT* and *IT* expression in the brain is limited to the hypothalamus, especially in the POA region, which is the center of sex-related genes. Surprisingly, *VT* mRNA is expressed in the dorsal and medial telencephalic regions of the cichlid fish (*Astatotilapia burtoni*) (29). These results indicate that the neurohypophysial hormones regulate sexual behavior through interactions with other key genes involved in reproductive behavior.

VT plays a role in regulating the sexual behavior of both male and female fishes. VT controls oocyte maturation and ovulation in female catfish by stimulating estrogen secretion and inhibiting progestin steroids (14). In male zebrafish, VT stimulates courtship behavior and reproductive physiology (30). In addition, VT plays a role in regulating aggressive behavior in mudskippers (31). In ovoviviparous fishes, VT can induce premature parturition in the topminnow (*G. affinis*) and guppy (*P. reticulata*) (15). VT can also induce premature parturition in ovoviviparous fishes with male pregnancy, including in lined seahorses. We observed that after VT injection, pregnant seahorses performed parturition and continuously shook their bodies to let the seahorse fries hatch out from the brood pouch. Sex steroids, including estrogen, play an important role in regulating sex-role reversal behavior (32). In lined seahorses, serum estrogen levels increase during pregnancy and decrease during the post-pregnancy stage (33). In the present study, the mRNA levels of *GPER* increased significantly after VT injection. This finding is in agreement with the postparturition upregulation of *GPER* in big-belly seahorses (34). These findings demonstrate that sex steroids, such as estrogen, play important roles in the parturition of pregnant seahorses. Moreover, VT induces parturition in pregnant seahorses and brood pouch compression, a process that may be mediated by GPER.

In the lined seahorse, VT induces estrogen secretion by stimulating gonadotropin (FSHβ and LHβ) synthesis during the parturition stage. This finding suggests that VT regulates reproductive physiology through the HPG axis in lined seahorses. VT also downregulated the *PRL* mRNA expression during parturition in lined seahorses. PRL maintains the brood pouch in a proper condition to support embryonic development in seahorses. In addition, PRL hormone levels are higher in the mating stage than in the parturition stage (16). These results indicate that VT is involved in the premature parturition of seahorses by upregulating estrogen secretion and downregulating PRL synthesis. Taken together, VT regulates the fish reproductive behavior *via* the HPG axis, in which the VT-stimulated gonadotropin synthesis and inhibited prolactin secretion in the pituitary regulate the estrogen secretion.

The premature release of embryos might be a stress response in pregnant seahorses. Insufficient nutrition, characterized by limited food consumption for a relatively long period, might promote premature parturition to release the embryos and keep them alive through VT stimulation. Studies have shown that VT is involved in the stress response; VT neurons innervate the corticotropic cells of the pituitary and influence adrenocorticotropin, which is secreted from the interrenal (5, 35). Pregnant seahorses may upregulate VT hormones in response to stress when suffering from nutritional deficiencies.

In conclusion, the present study reported the physiological role of neurohypophysial hormones in the reproductive behavior of ovoviviparous fishes with male pregnancy. Moreover, we demonstrated their roles in regulating parturition through the HPG axis. These findings suggest that VT regulates parturition behavior by upregulating serum estrogen levels. In addition, VT induces parturition through the membrane estrogen receptor (GPER). Our results shed light on the endocrinological mechanism of neurohypophysial hormones in regulating the reproductive behavior of teleosts.

## Data availability statement

The original contributions presented in the study are included in the article/**Supplementary Material**. Further inquiries can be directed to the corresponding author.

## Ethics statement

The animal study was reviewed and approved by the Animal research and Ethics Committees of the Chinese Academy of Sciences.

## Author contributions

HZ and QL designed the research. HZ and YL carried out the experiment. HZ, YL, and GQ analyzed the experiment data. QL provided lab space and equipment. All authors contributed to the article and approved the submitted version.

## Funding

The present study was supported by the Key Special Project for Introduced Talents Team of Southern Marine Science and Engineering Guangdong Laboratory (Guangzhou) (GML2019ZD0401, GML2019ZD0407), the Strategic Priority Research Program of the Chinese Academy of Sciences (No. XDA19060301), the National Science Foundation of China (No. 41976116, 41825013), the Youth Innovation Promotion Association CAS (2019337), and Guangdong Basic and Applied Basic Research Foundation (2021A1515011393) the Special Foundation for National Science and Technology Basic Research Program of China (2018FY100100), Science and Technology Planning Project of Guangdong Province, China (2020B1212060058).

## Conflict of interest

The authors declare that the research was conducted in the absence of any commercial or financial relationships that could be construed as a potential conflict of interest.

## Publisher’s note

All claims expressed in this article are solely those of the authors and do not necessarily represent those of their affiliated organizations, or those of the publisher, the editors and the reviewers. Any product that may be evaluated in this article, or claim that may be made by its manufacturer, is not guaranteed or endorsed by the publisher.

## Supplementary material

Supplementary Figure 1 Conserved synteny for the genomic region comprising *VT* and *IT* gene loci in teleosts. The gene organization and orientation in the genomic region containing the *VT* and *IT* genes were obtained from the Ensembl genome browser (http://www.ensembl.org).
