# Supplementary figures and images for: Identification of neurohypophysial hormones and the role of VT in the parturition of pregnant seahorses (Hippocampus erectus)
Source: Front Endocrinol (Lausanne). 2022 Jul 29;13:923234. doi: 10.3389/fendo.2022.923234 (PMC9372264; doi:10.3389/fendo.2022.923234)

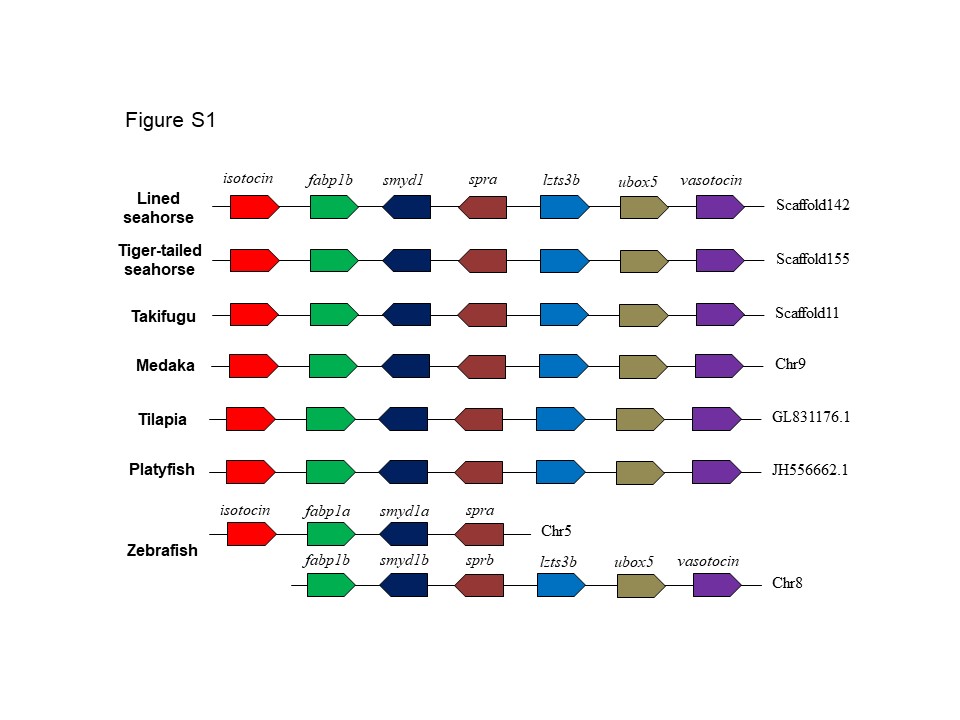

Supplement: Supplementary Figure 1 — Conserved synteny for the genomic region comprising VT and IT gene loci in teleosts. The gene organization and orientation in the genomic region containing the VT and IT genes were obtained from the Ensembl genome browser (http://www.ensembl.org). [file Image_1.jpeg]
